# Comparison of COVID-19 Vaccine-Associated Myocarditis and Viral Myocarditis Pathology

**DOI:** 10.3390/vaccines11020362

**Published:** 2023-02-05

**Authors:** Kamron Reza Hamedi, Gannett Loftus, Lawson Traylor, Richard Goodwin, Sergio Arce

**Affiliations:** 1 School of Medicine Greenville, University of South Carolina, 607 Grove Rd, Greenville, SC 29605, USA; 2 School of Medicine, University of South Carolina, 6311 Garners Ferry Rd, Columbia, SC 29209, USA; 3 Prisma Health Cancer Institute, Prisma Health System, 890 W Faris Rd Suite 320, Greenville, SC 29605, USA

**Keywords:** myocarditis, COVID-19, vaccine, congenital heart defect, fibrosis, ASIA

## Abstract

The COVID-19 pandemic has led to significant loss of life and severe disability, justifying the expedited testing and approval of messenger RNA (mRNA) vaccines. While found to be safe and effective, there have been increasing reports of myocarditis after COVID-19 mRNA vaccine administration. The acute events have been severe enough to require admission to the intensive care unit in some, but most patients fully recover with only rare deaths reported. The pathways involved in the development of vaccine-associated myocarditis are highly dependent on the specific vaccine. COVID-19 vaccine-associated myocarditis is believed to be primarily caused by uncontrolled cytokine-mediated inflammation with possible genetic components in the interleukin-6 signaling pathway. There is also a potential autoimmune component via molecular mimicry. Many of these pathways are similar to those seen in viral myocarditis, indicating a common pathophysiology. There is concern for residual cardiac fibrosis and increased risk for the development of cardiomyopathies later in life. This is of particular interest for patients with congenital heart defects who are already at increased risk for fibrotic cardiomyopathies. Though the risk for vaccine-associated myocarditis is important to consider, the risk of viral myocarditis and other injury is far greater with COVID-19 infection. Considering these relative risks, it is still recommended that the general public receive vaccination against COVID-19, and it is particularly important for congenital heart defect patients to receive vaccination for COVID-19.

## 1. Introduction

The COVID-19 pandemic has left its mark across the globe with a reported mortality rate of approximately 0.13% with around six million total COVID-19 deaths reported worldwide [1,2,3]. This prompted an unprecedented development cycle of a messenger RNA (mRNA) vaccine, the first of its kind used in the general public. While generally considered safe, concern about the long-term health risks and adverse effects have been raised due to its shorter development cycle. Since its approval for general use, reports of confirmed vaccine-associated myocarditis, particularly among young males, has prompted additional investigations into its cause, severity, and treatment [4]. However, studies regarding the long-term effects of COVID-19 vaccine-associated myocarditis are sparse given the low incidence and relatively recent distribution of the vaccine. Other vaccines have also had cases of vaccine-associated myocarditis, but longitudinal studies on heart function post-recovery are lacking [5]. The concern for long-term cardiac dysfunction becomes heightened in children and adults with congenital heart defects (CHD) as they are predisposed to cardiac fibrosis and other cardiomyopathies [6]. Studies regarding the effects of COVID-19 and its vaccine in this population are few. This review compares the pathways involved in viral myocarditis with those believed to be involved in vaccine-associated myocarditis, including COVID-19 vaccine-associated myocarditis, and relates this risk of myocarditis to the CHD patient population. The most important pathways in COVID-19 vaccine-associated myocarditis are likely prolonged and uncontrolled cytokine-mediated inflammation and autoantibody formation [7]. Myocarditis, regardless of cause, increases the risk for cardiac fibrotic remodeling that may persist despite resolution of myocarditis [8,9]. Many of the cytokine pathways, particularly transforming growth factor beta (TGF-β) and tumor necrosis factor alpha (TNF-α), are directly involved in stimulating tissue fibrosis [10,11]. Studies have been inconsistent regarding the risk of COVID-19 viral myocarditis in the CHD population, and studies regarding COVID-19 vaccine-associated myocarditis in this population are non-existent [12]. However, while some caution is warranted due to risk of cardiac damage, the risk of serious morbidity and mortality from COVID-19, including COVID-19 viral myocarditis, outweigh the risks of COVID-19 vaccine-associated myocarditis in the CHD population.

## 2. Viral Myocarditis

**Figure 1:**
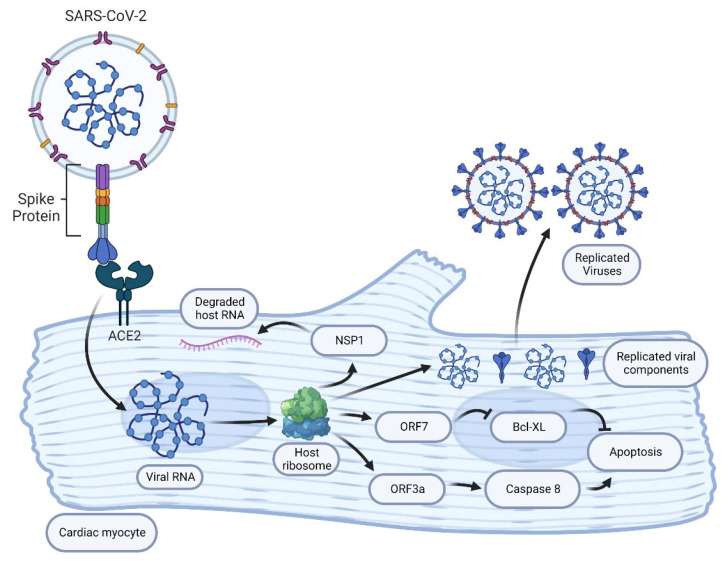
Direct myocyte damage from COVID-19 infection. The COVID-19 virus is brought into the cell when the spike protein binds to the ACE2 receptor. The viral RNA is exposed and then replicated by host ribosomes to produce new viral components. In the case of COVID-19, replicated viruses bud out from the host plasma membrane, rather than requiring cell lysis. Replication of the COVID-19 virus occupies host ribosomes and consumes host resources needed for normal cell function, leading to cell damage. Replication of viral RNA also produces non-structural protein 1 (NSP1) which degrades host RNA to allow more processing of viral RNA. Viral RNA production releases open reading frame 7 (ORF7) which inhibits B-cell lymphoma-extra-large (Bcl-XL), an inhibitor of apoptosis. Open reading frame 3a (ORF3a) is also produced and initiates apoptosis through activation of caspase-8. These open reading frames together initiate host cell apoptosis and inhibit pro-survival pathways. Figure created with BioRender.

Myocarditis is a potentially deadly condition most often caused by infectious diseases, though hypersensitivity reactions and autoimmunity have also been implicated [13,14,15]. Myocarditis affects as many as one and a half million people worldwide per year, with viral etiologies being the most common. Viral myocarditis is estimated to occur in 1–5% of viral infections, with the most common pathogens being enteroviruses, coxsackievirus, herpes, and parvovirus B19 [16]. Viral myocarditis is not uncommon among respiratory viruses, including influenza, and now severe acute respiratory syndrome coronavirus 2 (SARS-CoV-2), also called COVID-19 [17,18,19]. Symptoms of myocarditis most often include chest pain, fatigue, shortness of breath, or dyspnea and may present similar to congestive heart failure. It is not unusual for symptomatology to be severe enough to require hospitalization, but many cases are believed to be subclinical leading to underreporting after viral infections [16]. Treatment is largely symptomatic, focusing on maintaining oxygenation and cardiac function. Corticosteroids can control inflammation but otherwise have not been shown to improve mortality in myocarditis [16,20]. The prognosis of viral myocarditis is generally good with most patients recovering cardiac function back to baseline. However, long-term prognosis depends on the severity of presenting symptoms and severity of infection [16]. Viral myocarditis had a mortality rate of 19% from cardiac causes with those presenting with New York Heart Association (NYHA) stage one heart failure and no beta blocker therapy having the worst prognosis. The presence of scarring was also associated with increased risk of mortality, but a specific viral agent was not associated with increased mortality [21,22,23]. There are several main pathways which cause viral myocarditis: direct injury from infection of cardiac cells, direct injury to other cardiac tissues that disrupt cardiac function, inflammation from the immune response to infection and injury, and autoantibody formation in response to released intracellular material [17]. Direct damage typically occurs due to the normal viral lifecycle. As the virus replicates in the infected cell, native cell systems are commandeered, and the cell is eventually killed to release replicated virions [24]. In the case of an enveloped virus, such as COVID-19, lysis is not necessary for viral release. Instead, direct cellular damage is attributed to the dysregulation of normal cell function and apoptosis (Figure 1). Viral open reading frames (ORFs) are areas of viral genomes with potentially transcribable stretches of RNA. These ORFs do not always code for proteins and their functions are not well understood, but research continues to demonstrate their importance to viral function [25]. Apoptosis is attributed to the activity of ORF7 and ORF3a, which are both necessary for viral replication. ORF7 has been shown to inhibit anti-apoptotic B-cell lymphoma-extra-large (Bcl-XL) pathways, while ORF3a increases caspase-8 activity, a protease involved in the cleavage and activation of other caspases as part of the death-inducing signaling complex (DISC) (Figure 1) [26,27,28,29,30,31]. COVID-19 also disrupts host mRNA translation and increases mRNA degradation through non-structural protein 1 (NSP1) production (Figure 1) [32].

**Figure 2:**
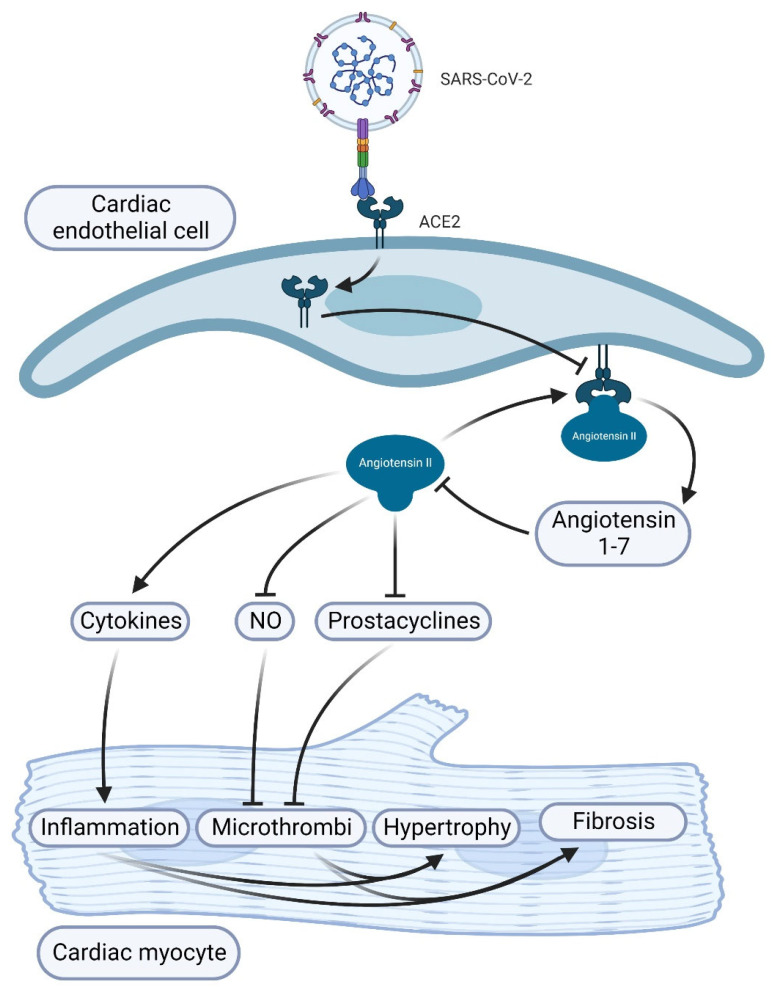
Indirect myocyte damage from COVID-19 infection. COVID-19 viruses enter endothelial cells when the spike protein binds to the ACE2 receptor. The receptor with virus is brought into the cell, leading to overall reduction in ACE2 receptor expression in the plasma membrane. ACE2 regulates angiotensin II activity when angiotensin II binds to the receptor, causing angiotensin II degradation and the production of angiotensin 1–7. Angiotensin 1–7 also acts to inhibit angiotensin II activity. Reduction of ACE2 receptor presence leads to decreased angiotensin II inhibition. Angiotensin II causes damage to cardiac myocytes through cytokine-induced inflammation and facilitating microthrombi formation through inhibition of vasodilators such as nitric oxide (NO) and prostacyclin. Inflammation and ischemic injury lead to myocyte hypertrophy and fibrosis. Figure created with BioRender.

However, only a few viruses, including COVID-19, directly infect cardiac myocytes [33,34,35,36]. Indirect viral damage to cardiac myocytes is caused by infection of surrounding cardiac endothelial cells or other associated cardiac tissue, the disruption of which causes cardiac damage and dysfunction [33,35]. In addition to physiologic dysfunction, infection of endothelial cells can have other indirect effects on cardiac myocytes depending on specific viral pathways. In the case of COVID-19, attachment to ACE2 receptors not only allows viral entry into the endothelial cell, but also downregulates ACE2 control of angiotensin II activity, which normally occurs through angiotensin II degradation and angiotensin 1–7 production (Figure 2) [37]. This excess angiotensin II causes increased inflammatory cytokine production and risk of thrombosis due to decreased nitric oxide (NO) and prostacyclin (Figure 2) [37]. Endothelial dysfunction has also been associated with myocardial hypertrophy and interstitial fibrosis, both of which are observed in viral myocarditis [37].

The most significant contributing pathways for myocarditis development are those from inflammation. Viral particles are recognized by toll-like receptors (TLR), nucleotide oligomerization domain (NOD)-like receptors (NLR), and intracellular pattern recognition receptors (PRR) [38,39]. Activation of TLR initiate pathways leading to the production of inflammatory cytokines including interleukin 1β (IL-1β), interleukin-6 (IL-6), TNF-α, and interferon gamma (IFN-γ). While there can be pathogen-specific cytokine patterns, many of the same cytokines are involved regardless of etiology [33]. TNF-α is one of the most commonly involved cytokines and is responsible for initiating apoptosis. Its pathways activate many caspases, which are protease enzymes required for apoptotic death signaling. TNF-α also decreases cardiac contractility by interfering with calcium release, thus further contributing to decreased cardiac function [33]. IL-6 is of significance because elevated levels denote a poor prognosis in viral myocarditis [40,41]. IL-6 plays several roles in the development of myocarditis, including decreasing cardiac contractility, increasing expression of adhesion molecules for natural killer cells (NK), lymphocytes, and macrophage recruitment, and mediates the development of autoimmunity [42,43]. The autoimmunity driven by IL-6 activity is believed to increase production of T-helper 17 cells (Th17) which have been strongly implicated in autoimmune myocarditis [42,44]. The recruitment of NK cells and macrophages mediates additional injury as these immune cells often attack healthy cells, along with infected cells [33]. IL-1β also mediates cardiac dysfunction similar to TNF-α by disrupting calcium signaling and cardiac contractility, while also mediating caspase-dependent and -independent apoptosis pathways [33]. The increased production of inflammatory cytokines, including IL-12 in particular, can lead to the activation of T-helper 1 cells (Th1) and the downregulation of T-helper 2 cells (Th2) [45,46]. Th2 cells aid in anti-inflammatory pathways, while Th1 produces additional inflammatory cytokines such as IFN-γ and recruits CD8+ T-cells [47]. These CD8+ T-cells also mediate cellular death, contributing to cardiac tissue damage and autoimmune reactions [33]. However, studies in mice have indicated that excess Th2 activity can lead to other forms of autoimmune myocarditis, indicating that activation of either the Th1 or Th2 phenotype can result in inflammation [47]. The development of cytokine storms is believed to be the major pathway of cardiac injury due to significantly increased Th1 activity and Th2 suppression mediated by high levels of IFN-γ, IL-6, IL-12, and IL-1β production [33,45]. Treatment with plasma exchange or rituximab, a monoclonal antibody used to deplete B-cells, has shown a reduction of IL-6 and TNF-α serum levels, adding support to the role of these cytokines in this disease process [48,49]. In addition to the production of antibodies, B-cells contribute to the production of IFN-γ when differentiated into B-effector-1-cells (Be1-cells). Type I IFNs have been observed in some forms of autoimmune myocarditis and are key mediators, along with IL-12, in the differentiation of Be1-cells [50,51]. TNF-α has been reported to be elevated in some COVID-19 patients, but this has not been consistent in all studies [33,45]. The final major pathway of viral myocarditis is autoimmune myocarditis, caused by damage to cardiac myocytes. Most commonly, as intracellular myosin is released by lysed myocardial cells, it is taken up and recognized by the immune system to produce anti-myosin antibodies leading to autoimmune myocarditis [52]. As previously mentioned, Th17 cells stimulated by IL-6 contribute to the development of autoimmune myocarditis by activating B lymphocytes to increase autoantibody production [33,36]. Autoimmune myocarditis has increased incidence in males, which has been attributed to the pro-inflammatory effect of testosterone [53]. Estrogen, which has anti-inflammatory properties, can decrease TNF-α levels, creating a protective effect in women, while testosterone can increase IL-1β expression and drive cardiac fibrosis [53].

Viral myocarditis is caused by the amalgamation of many of these pathways depending on the specific pathophysiology of the inciting virus. In the case of enteroviruses, a very important initial pathway is the direct infection and disruption of cardiac myocytes [33]. In addition to viral replication and evasion of the immune response, enteroviruses can disrupt calcium signaling, protein secretion, and endoplasmic reticulum function, severely disrupting normal cellular function [33]. Inflammatory cytokines and immune cell recruitment are also involved in enterovirus myocarditis. IL-1, IL-6, and TNF-α are induced in enterovirus infections and lead to the recruitment of NK cells, macrophages, Th1 cells, and B lymphocytes [35,54]. Anti-myosin autoantibodies formed in response to lysed cardiac myocytes are also observed in enterovirus myocarditis [33,35]. Another common culprit of viral myocarditis is influenza. Influenza is able to infect cardiac myocytes, which leads to apoptosis, though this is not believed to be the primary pathway involved in influenza viral myocarditis [33,34,35]. Influenza is also known to directly infect endothelial cells thus indirectly driving myocarditis development [33]. However, the primary pathway active in influenza viral myocarditis is believed to be IL-1β, IL-6, and TNF-α dependent [33,35].

Another etiology of myocarditis linked to viral infections is systemic inflammatory or autoimmune syndromes. One of the most common syndromes associated with viral infections that causes cardiac dysfunction and myocarditis is Kawasaki disease in children [55]. While Kawasaki disease is primarily associated with coronary artery aneurisms from vasculitic post-streptococcal infection, it has been often associated with Epstein-Barr virus and adenoviruses, and does cause myocarditis [56,57,58]. Kawasaki is associated with systemic inflammation and elevated levels of IL-1β and TNF-α and vasculitis resulting from immunoglobulin A (IgA) antibody deposition in the coronary arteries [57]. There is also a hypothesized genetic predisposition as the disease is most frequently observed in patients of Asian descent. One potential genetic predisposition suggested is a single-nucleotide polymorphism (SNP) in the inositol 1,4,5-trisphosphate 3-kinase C (ITPKC) gene which effects the calcium/nuclear factor of activated T-cells (NFAT) pathway, leading to increased IL-1β production [59]. IL-1β increases the differentiation of CD8 T-cells contributing to the vascular damage seen in Kawasaki [56,60]. Another genetic abnormality is the increased CD40 ligand (CD40L) expression on CD4 T-cells, leading to hyperproduction of inflammatory cytokines, and is associated with increased risk of coronary artery aneurisms [56]. Ultimately this hyperproduction of cytokines and deposition of IgA-complement 3 (C3) complexes causes vascular damage associated with Kawasaki [56]. A closely associated syndrome is multisystem inflammatory syndrome in children (MIS-C) which has been associated with COVID-19 infection as a post-viral syndrome. Recent reports have identified the same type of injury in adults, called multisystem inflammatory syndrome in adults (MIS-A). This syndrome has also been associated with myocarditis and cardiac dysfunction [61]. Symptoms can be very similar to Kawasaki, but there is an increased incidence of myocarditis compared to coronary artery injury [62]. MIS-C depends on IL-1β activity to cause the systemic cytokine storm associated with the syndrome [62,63]. Some studies have also reported decreased levels of C3 and complement 4 (C4), suggesting complement involvement in vasculitis pathophysiology similar to Kawasaki [62]. Due to the similarities in symptoms, causes, and pathways, it has been suggested that Kawasaki, MIS-C, and MIS-A should be categorized as different points in the spectrum of a common disease syndrome [62,64].

Myocarditis in COVID-19 infection has contributed to the severity and mortality of the disease in some patients. While COVID-19 is primarily a respiratory virus, infection affects other organs, including the heart, leading to symptomatology and, in some cases, death [65]. Arrythmias, which occur in 78% of myocarditis patients, have been reported in as many as 7% of cases in some COVID-19 cohorts and were attributed to 44% of ICU admissions for COVID-19 infection [65]. In a Chinese cohort study, up to 17% of hospitalized COVID-19 patients showed evidence of acute cardiac injury. Within this cohort, 22% of COVID-19 patients requiring ICU admission showed evidence of cardiac injury compared to 2% of patients not admitted to the ICU. Of those who died from COVID-19 infection, 59% showed signs of acute cardiac damage [66]. Other studies demonstrated acute cardiomyopathy in 23% of hospitalized COVID-19 patients, though this did not account for preexisting heart failure [66,67]. These studies demonstrated that 51.9% of patients who died from COVID-19 infection showed signs of heart failure as well [66,67]. A large meta-analysis compared the risk for myocarditis across approximately 58 million patients (55.5 million COVID-19 vaccinated patients and 2.5 million COVID-19 infection patients) across 22 studies, ten of which were specific to determining the risk of myocarditis in COVID-19 infected patients. This analysis found that the relative risk of myocarditis in COVID-19 infection was 14.82 [68]. Using the data presented in this analysis, 134,785 out of 2.4 million patients diagnosed with COVID-19 also met the qualifications for myocarditis [68]. A Centers for Disease Control (CDC) Morbidity and Mortality report also found a relative risk of 15.7 for the development of COVID-19 infection-associated myocarditis compared to non-COVID-19 patients [69].

Many cohort and case studies have recorded incidents of cardiac involvement and myocarditis among hospitalized COVID-19 patients, including those with severe COVID-19 infection. One study estimated that COVID-19 myocarditis had a prevalence of 2.4 per 1000 hospitalized COVID-19 patients but increased to 4.1 per 1000 when unconfirmed myocarditis cases were included [70]. Of the confirmed cases of COVID-19 myocarditis, 70% required ICU admission [70]. Another cohort study of 1721 patients hospitalized with COVID-19 found that 0.7% of patients were diagnosed with myocarditis [71]. One large metanalysis of over 70,000 patients demonstrated variation in the development of myocarditis between the general COVID-19 patient population and those admitted to the ICU. Only 0.1% of the general COVID-19 patient population developed myocarditis, while in the ICU 0.5% were diagnosed [72]. This is consistent with a CDC Morbidity and Mortality report which stated that COVID-19 patients had a 0.146% risk of being diagnosed with myocarditis [69]. Myocarditis has contributed to approximately 7% of COVID-19 fatalities in some studies, with cardiac failure contributing to 33% of fatalities in combination with respiratory failure [65,66,73]. This is consistent with other postmortem cases demonstrating evidence of myocarditis in 7% of the patients [73].

The overall mortality rate of patients with COVID-19 myocarditis has been reported to be 14% by some studies [73]. Others have reported 6.6% mortality across all COVID-19 myocarditis patients, but it increased to 15.1% when associated with pneumonia [70]. This is similar but slightly lower than the estimated 19% mortality for viral myocarditis from other viruses [16]. While these data represent myocarditis in severe COVID-19 leading to ICU admission or death, it highlights the role this condition can play in the severity and mortality of COVID-19 infection. While the absolute risk of developing COVID-19 infection-associated myocarditis is low and the mortality rate slightly lower than other sources of viral myocarditis, COVID-19 infection showed a significantly increased relative risk of developing myocarditis [16,68,69,72]. The risk of developing myocarditis from COVID-19 is also increased in patients with previous cardiovascular comorbidities. This is believed to be caused by the increased expression of ACE2 in these patients, leading to greater risk of cardiac infection and myocarditis [73]. Children are at decreased risk of COVID-19 infection and typically have a milder disease course. However, severe infection requiring ICU admission for pediatric COVID-19 infection showed that 83% had comorbid conditions, many of which were simply defined as “medically complex” [74]. There may be some difference in the development of myocarditis from COVID-19 infection depending on the COVID-19 variant. The recent omicron COVID-19 variant has been observed to have increased transmission with less severe infection and fewer hospitalizations [75,76,77,78,79]. Little is known regarding the effect of the omicron COVID-19 variant on cardiac function; however, a case study was reported regarding two patients presenting in January 2022 with the polymerase chain reaction (PCR) confirmed omicron variant, and an MRI confirmed myocarditis. Both patients had received both initial BNT162b2 vaccine doses in addition to a BNT162b2 booster in Augusts 2021. The first patient, a 40 year-old male, recovered with the administration of ibuprofen and colchicine. The second patient, a 60 year-old male, has recovered but did require placement of an implantable cardioverter-defibrillator (ICD) [79]. As the omicron COVID-19 variant has been shown to have decreased severity, but still capable of causing myocarditis, future research is warranted to determine the prevalence of COVID-19 myocarditis with these new variants [80]. This epidemiologic data showed the risk that development of myocarditis in COVID-19 infection has on patient outcomes.

## 3. Vaccination-Induced Autoimmunity and Myocarditis

As a result of the COVID-19 pandemic, the benefits and risks of the COVID-19 vaccines have become an important topic. While predominantly a viral respiratory infection, COVID-19 has effects on multiple organ systems, including the cardiovascular system. As mentioned before, the mortality rate has been reported around 0.13% with an estimated six million reported deaths [1,2,3]. The accelerated production and distribution of these vaccines was justified, but it has raised questions about the long-term side effects, which have now been reported. While generally considered safe, the COVID-19 vaccines have risks and benefits like all other vaccines. The mRNA vaccines have been associated with higher rates of reactogenicity, with the most common reactions including local injection site reactions, such as redness and pain, and systemic reactions, such as body aches and fever [81,82,83]. Fatigue was reported in 30.9% of patients after receiving the first dose of either the Pfizer or Moderna mRNA vaccine and 53.9% after the second dose [81]. Fever was reported as 8.6% and 29.5% after the first and second doses, respectively [81]. However, while in most patients these symptoms were mild and short-lived, the immune system can cause inflammatory damage through the development of hyperinflammatory syndromes [84]. A rare but important sequalae of hyperactive inflammation is vaccine-associated myocarditis, which has the potential to cause or worsen preexisting heart damage and, in exceedingly rare cases, cause death [4,7,85,86,87,88,89,90]. Chest pain was noted in 8% of vaccine recipients, while dyspnea was noted in as many as 15%, both of which are associated with myocarditis.

Safety reports have shown that cases of myocarditis after COVID-19 vaccination were as many as 4.4 events per million recipients [91]. Other reports have stated that the rate of vaccine-associated myocarditis was 105.9 cases per million doses of the COVID-19 mRNA vaccines in males 16–17 years old, greater than expected rate in non-vaccinated populations [92,93]. One safety report showed that the risk ratio for vaccine-associated myocarditis was 3.24 compared with non-vaccinated populations [94]. A large meta-analysis found that the relative risk of COVID-19 vaccine-associated myocarditis was two compared to 15 for COVID-19 infection-associated myocarditis, a seven-fold increase in risk for infection associated myocarditis [68]. This is consistent with a second meta-analysis with 58.6 million patients which showed a relative risk for myocarditis of 2.36. This same analysis demonstrated greater risk in males, patients under the age of 40, and those who received two doses of the Moderna mRNA-1273 vaccine [95]. A higher prevalence in men at 61% was observed, which is consistent with other studies, though in some reports this was shown to be as high as 94% [68,96,97]. Important studies using adverse-event reporting systems identified the greatest risk for vaccine-associated myocarditis in young adult males after the second vaccine dose, particularly if given in less than a 30 day interval [89,90,92,98,99]. Another large meta-analysis (Khan el at, 2022) stated that the higher incidence of vaccine-associated myocarditis was observed after two doses of the Pfizer BNT162b2 mRNA vaccine [96]. However, another study (Patone et al., 2022) demonstrated that the highest rate of vaccine-associated myocarditis was observed after the second dose of the Moderna mRNA-1273 vaccine with a risk of 11.76% compared to Pfizer’s 1.57% [100]. This study also showed that the excess events of myocarditis after the second dose of the mRNA-1273 vaccine in young males was higher than those observed in COVID-19 infections [100]. The higher incidence of myocarditis after the Moderna mRNA-1273 is consistent with findings from other large studies; however, the observation that the excess rate of myocarditis after COVID-19 vaccination was higher than after COVID-19 infection is not consistent with other meta-analyses [68,95,101]. While COVID-19 infection-associated myocarditis can still occur after vaccination, Patone et al. showed that the number of excess cases dropped from 35 per 1,000,000 to 23 per 1,000,000 after vaccination [100]. Additionally, 96% of those with COVID-19 vaccine-associated myocarditis required hospitalization [92]. While rare, this indicates that the increased rate of myocarditis can be attributed to the COVID-19 vaccine and has the potential to be serious. As compared to the estimated mortality from COVID-19 infection-associated myocarditis, no consensus on mortality rate could be found for COVID-19 vaccine-associated myocarditis. Deaths have been reported, but are rare. One retrospective study by Mevorach et al. reported only one death out of 304 patients with vaccine-associated myocarditis [99]. Another large study by Witberg et al. likewise demonstrated one death in a patient with cardiac comorbidities out of 54 patients with myocarditis [97]. Lai et al. observed a mortality rate of <1 per 10,000 per day in a large study comparing the outcomes of 104 COVID-19 vaccine-associated myocarditis patients with a viral myocarditis counterpart [102]. In addition to improved mortality, vaccine-associated myocarditis typically has decreased severity of disease compared to COVID-19 infection-associated myocarditis with clinical improvement seen after receiving pain management as reported by Oster et al. [92]. Lai et al. also observed decreased severity of illness in their study. Taken together, these studies showed that while COVID-19 vaccine-associated myocarditis is a significant adverse event, it occurs less often and is less severe than COVID-19 infection-associated myocarditis.

The suspected pathways involved in vaccine-associated myocarditis are varied and highly dependent on the individual vaccine. Cytokine hyperstimulation is a general term that encompasses responsible pathways that are vaccine and individual specific. The causes of cytokine hyperstimulation are believed to be due to differences in genetic variants of the cytokines, cytokine receptors, and/or the types of cells involved in the specific immunologic pathways accounting for variable patient symptoms because of the elevated cytokine levels. Even when viral components are attenuated to have significantly reduced immunogenicity, these genetic differences can modify the reactivity to the vaccine in some subjects, causing adverse inflammation and even organ damage, resembling true infection [103]. While the pathways are varied, many can either be classified as type IV hypersensitivity reactions, or are analogous to them, due to the involvement of T-cells, accessory cells, and specific inflammatory cytokines [103,104,105]. Which subtypes of type IV reactions are involved is highly dependent on which pathways are activated.

**Figure 3:**
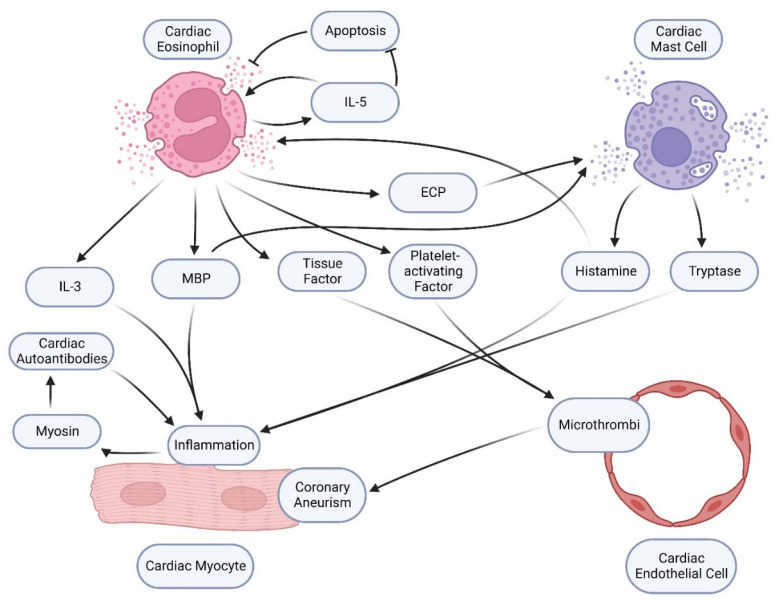
Cardiac damage pathways from eosinophilic myocarditis. Cardiac eosinophil degranulation causes direct damage to cardiac myocytes through the release of interleukin-3 (IL-3) and major basic protein (MBP), causing inflammation. Interleukin-5 (IL-5) is released and recruits more eosinophils and inhibits apoptotic pathways, increasing the total eosinophil load locally in the cardiac tissue. The MBP, along with eosinophil cationic protein (ECP), also causes degranulation of cardiac mast cells. This releases histamine and tryptase, additional mediators of myocyte inflammation. Histamine has the secondary function of recruiting additional eosinophils to the area. Tissue factor and platelet-activating factor release from eosinophil degranulation, leading to platelet aggregation and microthrombi in cardiac endothelial cells, causing indirect damage to cardiac myocytes and increasing the risk for coronary aneurisms. Inflammation and injury to cardiac myocytes releases intracellular myosin which can be recognized by the immune system which develops myosin autoantibodies, compounding myocyte inflammation and injury. Figure created with BioRender.

Another similar reaction is eosinophilic myocarditis. The specific mechanism of cardiac damage caused by eosinophilic myocarditis is variable as well [106]. Some studies showed that eosinophil recruitment can cause direct damage to myocytes, but other studies suggest this may not be a consistent component of this condition [107]. Regardless of the importance of direct damage, the cytokines and proteins produced by eosinophils are known to be cytotoxic. These include eosinophil major basic protein (MBP), interleukin-3 (IL-3), and interleukin-5 (IL-5) (Figure 3) [108,109]. IL-5 recruits additional eosinophils and exerts an anti-apoptotic affect, increasing local eosinophil infiltrates and compounding the damage (Figure 3) [110,111]. This is consistent with a type IVb hypersensitivity reaction [86,105,112]. Downstream, MBP and eosinophil cationic protein (ECP) from eosinophilic degranulation induce histamine and tryptase release from cardiac mast cells, leading to further tissue damage also consistent with type IVb hypersensitivity reactions (Figure 3) [105,112]. Histamine released by cardiac mast cells recruits additional eosinophils, resulting in a positive feedback loop (Figure 3) [113]. Eosinophils have also been found to cause microthrombi from the release of tissue factor (TF) and platelet-activating factor (PAF), two procoagulant factors which increase the risk of tissue ischemia and necrosis (Figure 3) [114]. Vasculitis syndromes, such as Kawasaki disease, while usually associated with post-viral infection, have been observed after vaccination for rotavirus and COVID-19 and is a good model for the vascular damage caused by eosinophilic myocarditis [115,116]. Similar to Kawasaki disease, untreated eosinophilic myocarditis can lead to coronary aneurisms [117]. While the involvement of T-cells and cytokines suggests a type IV hypersensitivity reaction, recent studies suggest Kawasaki and similar conditions are type III hypersensitivity reactions [118]. Type III reactions rely on the formation and deposition of antigen-antibody complexes which activate complement, leading to inflammation and tissue damage [105,119]. A final pathway of eosinophilic myocarditis that is currently theoretic is the release of autoantigens from cardiac tissue secondary to tissue damage from the activation of the previously mentioned pathways (Figure 3) [120]. While awaiting experimental proof, this theory proposes that the inflammation and damage caused by eosinophilic myocarditis releases intracellular components that can themselves become autoantigens [120,121]. This has been observed in other autoimmune conditions, including autoimmune myocarditis [121,122,123]. In this case, inflammation and cellular damage releases myosin which acts as an autoantigen for the priming of autoimmune processes [124,125,126]. These autoantigens are recognized by the immune system with subsequent autoantibody production and cardiac tissue damage. Myosin autoantibodies from other forms of myocarditis demonstrated antibody-mediated cyclic adenosine monophosphate (cAMP)-dependent protein kinase A (PKA) activity through binding to beta-adrenergic receptors consistent with type II hypersensitivity reactions [105,127,128,129,130]. Overstimulation of cardiac beta receptors, including antibody-mediated stimulation, has resulted in cardiac myocyte death and development of cardiomyopathy in other studies [131,132].

Similar to the eosinophilic myocarditis autoantigen pathway is molecular mimicry. While the antigens used in vaccines are carefully chosen to avoid cross reactivity, some have molecular similarities with normal human tissue. The antibodies formed against these antigens have the risk of recognizing and damaging healthy tissue that is erroneously identified as foreign [133]. Depending on the degree of similarity, the risk for molecular mimicry may be partially genetic due to a dysfunction in central or peripheral tolerance [133]. Central tolerance is the mechanism by which the body removes lymphocytes from the bone marrow and thymus that could react to native tissue, while peripheral tolerance keeps in check self-reactive lymphocytes that either survived central tolerance or expanded in the periphery [121,134]. If these protective systems are dysfunctional, the risk that an antigen could recruit a lymphocyte with self-reactive properties increases. Even if central tolerance is not impaired, B-cell autoimmunity can still occur due to clonal expansion in lymph node germinal centers [135,136]. Clonal expansion involves significant somatic mutations in IgV region genes which drastically increases the risk of self-reactivity [121]. Intracellular autoantigens are often not involved in central tolerance which also creates a way for self-reactive lymphocytes to escape normally functioning central tolerance mechanisms [137]. A final common pathway for vaccine-associated myocarditis is autoimmune/inflammatory syndrome induced by adjuvants. In this case, the vaccine antigen itself is not involved in the inappropriate immune reaction. Instead, the adjuvant, a component added to vaccines to stimulate the innate immune system, creates a hyperactive response [138]. The active pathways involved in autoimmune/inflammatory syndrome induced by adjuvants (ASIA) are as varied as the adjuvants suspected in the development of this condition. These range from molecular mimicry, hyperstimulation of the immunogenic pathways, or toxicity of the adjuvant due to slowed or impaired elimination. In the case of aluminum, an adjuvant commonly used in vaccine formulations, its longevity in the body causes chronic immune system stimulation and is a risk for toxicity [138,139]. Newer adjuvants may also act as TLR agonists which, when hyperstimulated, could lead to excessive immune responses [140]. Often adjuvants are antigens themselves. ASIA is believed to encompass a wide variety of specific autoimmune or autoinflammatory conditions with analogues including Sjogren syndrome and sarcoidosis [141]. The criteria defining ASIA revolve primarily around induction of symptoms after exposure and resolution after removal of the antigen, with the presence of autoantibodies or adjuvant antibodies considered as minor criteria [142]. Certain genetic predispositions have also been noted with abnormalities in the human leukocyte antigen-DRB1 (HLA-DRB1) and Protein Tyrosine Phosphatase Non-Receptor Type 22 (PTPN22) genes [142]. These indications of genetic predisposition are consistent with the increased observation in young and otherwise healthy patient populations.

The COVID-19 vaccines are not the first vaccine associated with myocarditis. Rare cases have been reported with the pneumococcal, hepatitis B, smallpox, and human papilloma virus vaccines, and some formulations of the influenza vaccine [5,85,143,144]. Sympathetic dysregulation, cross-reactivity of immunogenic components, and genetic sensitivities of the immune system have all been implicated in its development [145,146]. In the case of the smallpox vaccine, a dysregulated inflammatory response due to small genetic variations in interleukin-4 (IL-4) leads to the adverse effects seen in some patients post-vaccination [5,146,147,148]. IL-4 is produced by Th2 cells, stimulates Th2 cell differentiation, and is active in anti-inflammatory pathways that attenuate inflammatory macrophage responses [149]. In the variants believed responsible, IL-4 is either less available or less effective, leading to increased signaling by macrophages and recruitment of neutrophils. Increased neutrophils result in tissue damage and inflammation observed in smallpox vaccine adverse reactions [146]. Other genetic variants with increased IL-4 activity showed decreased inflammatory responses, adding further evidence to the above observation. Genetic variants of interleukin-18 (IL-18) and interleukin-1 (IL-1) with increased sensitivity were also elevated in some post-vaccine immune responses [5]. Influenza vaccines are far less likely to cause myocarditis, but rare reports have been made [145,148,150,151]. Current evidence favors a hyperactive response to an adjuvant, consistent with ASIA. The adjuvant MF59, commonly used in influenza vaccines, has known immunogenicity and may account for the rare instance of ASIA post-influenza vaccine [152,153]. MF59 is an oil-in-water emulsion, which is taken up by antigen presenting cells (APCs) and stimulates the recruitment of additional monocytes, macrophages, and neutrophils [139]. This recruitment of APCs both creates an environment more likely to react to the vaccine antigen and acts as a positive feedback loop for MF59 where recruited APCs take up more MF59 and recruit increasing numbers of APCs [139]. MF59 is still used in influenza vaccines because of increased cross-reactivity between influenza strains when included in the vaccine [154]. While not associated with symptomatology, studies have found that the influenza vaccine can increase antinuclear antibody (ANA) production transiently. The human papillomavirus (HPV) vaccine has been associated with rare cases of cardiac damage and death as well [155]. A suggested mechanism is molecular mimicry between the viral antigen and multiple types of cardiac tissue. Titin, a component of striated muscle including cardiac myocytes, has an overlap of nine viral pentamers which could lead to cross reactivity [155,156]. When targeted by autoantibodies, titin has been shown to contribute to ventricular cardiomyopathy [157]. Rare cases of myocarditis have been associated with the hepatitis B vaccines [143,152]. Tissue samples showed eosinophilia consistent with eosinophilic myocarditis, the pathways of which we have previously discussed. What specific component of the vaccine triggers this eosinophilic reaction is unknown [143].

Due to the public prominence of the COVID-19 vaccines, much interest has been raised regarding the association of the vaccines with myocarditis. Several different mechanisms have been implicated in the development of COVID-19 vaccine-associated myocarditis [158]. Similar to the hepatitis B vaccine, the COVID-19 vaccines have shown evidence of eosinophilic myocarditis in some patients [159,160]. Cytokine dysfunction and autoantibodies have been observed as well, though these have not been consistent [85,93,161]. While COVID-19 mRNA in vaccines is modified to reduce the response by dendritic and TLR expressing cells, some patients with genetic variants in IL-6 may still mount enough of a cytokine response to cause inflammatory damage similar to an actual COVID-19 infection [7,93,162,163]. These cytokines, such as IL-6, IL-1, and TNF-α, are observed in severe COVID-19 infections and have been associated with post-infectious autoimmune reactions [163]. The COVID-19 vaccines can activate the same pathways that cause MIS-A and acute COVID-19 cardiovascular syndrome (ACovCS), which is associated with decreased left ventricular function without history of coronary artery disease, as well [4,164]. The exact mechanism of ACovCS is not well understood, but studies suggest that cardiac damage is mediated primarily by cytokine release syndromes initiated by B- and T-cell activation, rather than direct cardiac tissue damage [162,165]. High levels of IL-6 and IFN-γ observed post-COVID-19 vaccination strengthen this assumption as these cytokines, particularly IL-6, predominate in cytokine storms post COVID-19 infection [48,49]. TNF-α and IFN-y are both inflammatory cytokines also believed responsible for the cytokine storm associated with severe COVID-19 infection [48,49,163,165]. The COVID-19 mRNA vaccines occasionally have double-stranded mRNA contaminants which are potent inducers of TNF-α and IFN-y [166]. The presence of autoantibodies in some patients suggests that molecular mimicry with the COVID-19 spike protein produced by the mRNA of the vaccine is involved [7,85,93]. Some of these autoantibodies can cross-react with α-myosin, creating a link with direct cardiac tissue damage [162,167]. The exact cause of this molecular mimicry is not understood; however, both the COVID-19 spike protein and α-myosin share an alpha helical coiled coil structure which could be responsible [168]. This same shared coiled coil structure is believed to cause molecular mimicry between cardiac myosin and group A Streptococcal epitopes [169]. Unlike the influenza vaccine, COVID-19 vaccine-associated myocarditis has a higher predominance in men possibly due to the inhibition of anti-inflammatory processes in males [7,53,85,170]. A recent study found that COVID-19 vaccinated patients had similar T-cell and antibody profiles regardless of the presence of myocarditis, suggesting these were not involved in myocarditis development. However, increased levels of unbound spike protein were found in those who developed myocarditis after COVID-19 vaccination, possibly from a defect in spike protein clearance in these patients [171]. COVID-19 spike proteins can cause cardiac tissue damage by decreasing ACE2 expression and increasing the permeability of the endothelial cell barrier [171]. The sample size of this study was small, however, and would need validation in larger patient populations. Regardless of the cause, COVID-19 vaccine-associated myocarditis is an important consideration as it currently accounts for a significant amount of vaccine associated myocarditis. While this may be an artificially high observation due to the number of COVID-19 vaccines distributed and the scrutiny it has received, it is still a concern worth investigating due to its potentially serious adverse effects.

## 4. Effects of Myocarditis on the Heart

Once myocarditis develops, several different outcomes can occur depending on the mechanism of injury. In many cases, vaccine-associated myocarditis is self-limited and will resolve on its own. In more severe cases, or in viral myocarditis, sustained damage leads to disease progression. When continued cytokine production or autoantibodies from molecular mimicry is involved, the increased damage to the heart ultimately leads to dilated cardiomyopathy [125]. If the injury to the heart is severe, the molecular debris released by the lysed cardiac tissue may lead to additional cytokine or autoantibody recruitment and cardiac impairment [125]. Thankfully, vaccine-associated myocarditis, which itself is rare, usually occurs in young patients and causes less severe myocarditis that resolves quickly and rarely leads to mortality [172]. However, studies in recovered patients under 40 with no known cardiovascular comorbidities showed cardiac imaging with myocardial fibrosis and necrosis with unknown significance for longer term function [173,174]. This is concerning as residual myocardial fibrosis in other forms of myocarditis increases the risk for future cardiac pathology [8,9]. Due to resolution of symptoms and abnormal biomarkers, longitudinal imaging with cardiac magnetic resonance imaging (MRI) of recovered COVID-19 vaccine-associated myocarditis has not been done by any major studies but may be warranted due to risk of later complications [175].

**Figure 4:**
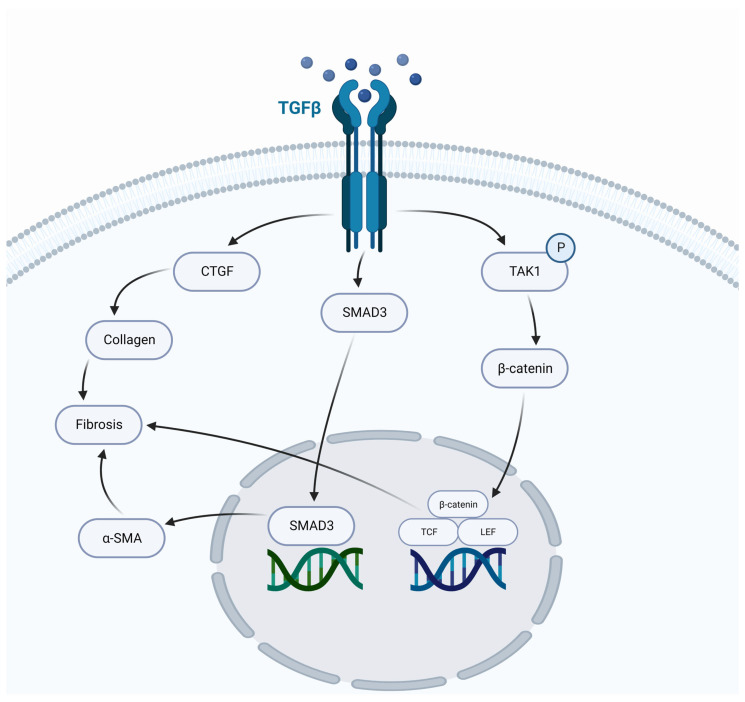
TGF-β pathways in cardiac fibrosis. Transforming growth factor beta (TGF-β) acts through several pathways to induce fibrosis in cardiac tissue. TGF-β induces the production of connective tissue growth factor (CTGF) which increases collagen production, ultimately ending in increased fibrosis. The wingless-related integration site (Wnt) pathway activates transforming growth factor-β activated kinase (TAK1), which auto-phosphorylates to increase β-catenin activity. β-catenin translocates to the nucleus where it acts as a transcription factor with T-cell factor (TCF) and lymphoid enhancer factor (LEF) to increase transcription of fibrogenic target genes. The traditional TGF-β pathway increases mothers against decapentaplegic 3 (SMAD3) activity, which is also a transcription factor that increases production of alpha smooth muscle actin (α-SMA), a protein observed in mature fibroblasts, and induces production of tissue fibrosis. Figure created with BioRender.

The pathway by which myocarditis stimulates fibrosis is an intricate network of immunological cascades that can have many inciting factors. In the case of coxsackieviruses, profibrotic cytokines such as TGF-β, TNF-α, IL-1, and IL-4, had elevated activity [176]. TGF-β is a strong mediator of cardiac remodeling through increased production of extracellular matrix proteins [177,178]. TGF-β also stimulates connective tissue growth factor (CTGF), which is associated with increased accumulation of collagen (Figure 4). This association is strengthened by CTGF being present in ischemic damage, leading to fibrotic cardiac remodeling [177,178,179,180]. Another pathway used by TGF-β to mediate fibrosis is the wingless-related integration site (Wnt) signaling pathway [179,181]. This is accomplished by autophosphorylation of transforming growth factor-β activated kinase 1 (TAK1), the blockade of which caused decreased Wnt-pathway products such as β-catenin in mice (Figure 4) [10]. β-catenin, once stabilized, becomes a transcription factor that binds to T-cell factors (TCFs) and lymphoid enhancer factors (LEFs) to increase transcription of Wnt target genes (Figure 4) [181]. When β-catenin is interrupted in mice models, decreased fibrosis has been noted, linking β-catenin-dependent Wnt pathways with myocardial fibrosis [10]. The traditional mediators of TGF-β signaling, mothers against decapentaplegic (SMAD) pathway, have also been linked to fibrosis. Increased Smad3 promotes alpha-smooth muscle actin (α-SMA) transcription, an important marker of mature myofibroblasts which are responsible for the carrying out the fibrotic myocardial remodeling (Figure 4) [178,180,182]. While there have been no studies assessing the activity of TGF-β in COVID-19 vaccine-associated myocarditis, the activity of TGF-β was increased in mouse models of autoimmune myocarditis called experimental autoimmune myocarditis [183]. This mouse model causes an autoimmune disorder targeting cardiac myosin. While it is not a perfect analogue for COVID-19 vaccine reactions, one proposed mechanism mentioned earlier is the formation of autoantibodies to α-myosin due to molecular mimicry with COVID-19 spike protein mRNA [158,183,184]. Elevated TGF-β is also implicated in autoimmune idiopathic pulmonary fibrosis (IPF) as a fibrotic biomarker in the condition [185]. This warrants further study to determine how much a role, if any, TFG-β has in COVID-19 vaccine-associated myocarditis and its potential long-term adverse side effects.

TNF-α is another active inflammatory cytokine that has been found elevated in mice cardiac tissue with myocarditis and has been implicated in autoimmune myocarditis [11,186]. TNF-α can also have protective antiviral effects [187]. This dichotomy suggests that while a moderate TNF-α response may be beneficial by aiding the elimination of viral pathogens, excessive response could lead to tissue damage out of proportion to its protective effects. This suggestion is strengthened as the level of TNF-α expression has been correlated with the degree of cardiac damage from myocarditis regardless of source [188,189].

**Figure 5:**
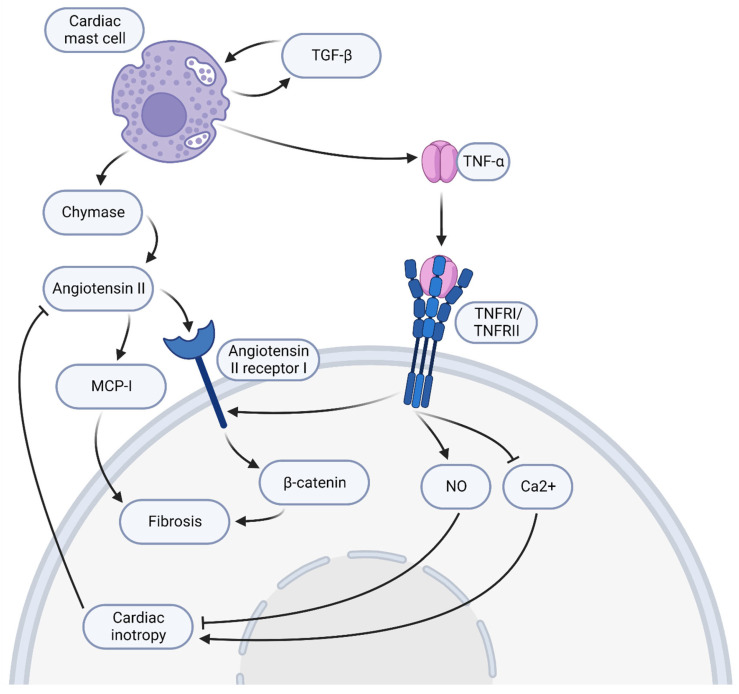
TNF-α pathways in cardiac fibrosis. Tumor necrosis factor alpha (TNF-α) binds to tumor necrosis factor receptors I and II (TNFRI and TNFR II) which can initiate several pathways that contribute to cardiac tissue fibrosis. TNFRI increases the expression of angiotensin II receptor I, which, when bound to angiotensin II, upregulates the β-catenin pathway to fibrosis. TNFRI and TNFRII upregulate nitric oxide (NO) production while decreasing Ca^2+^ fluxes. NO decreases cardiac inotropy while the inhibition of Ca^2+^ fluxes inhibits positive cardiac inotropy, collectively causing decreased cardiac inotropy and upregulating angiotensin II activity. Angiotensin II promotes macrophage chemotactic protein I (MCP-1) production to increase fibrosis in addition to the β-catenin pathway. Transforming growth factor beta (TGF-β) interacts with these pathways through the recruitment of cardiac mast cells. Mast cell degranulation releases additional TFG-β as well as TNF-α and chymase. Chymase converts angiotensin I to angiotensin II, increasing those associated pathways. Figure created with BioRender.

TNF-α binds two receptors on cardiac myocytes, tumor necrosis factor receptor I (TNFRI) and tumor necrosis factor receptor II (TNFRII) (Figure 5). TNFRI is responsible for initiating fibrosis from many causes by increasing renin-angiotensin activity and the expression of angiotensin II receptor 1 (Figure 5) [54]. The renin-angiotensin-aldosterone system (RAAS) is known to cause fibrosis and cardiac remodeling; hence angiotensin converting enzyme inhibitors (ACE)/angiotensin receptor blockers (ARBs) are a part of goal directed therapy in the treatment of heart failure [190,191]. TNF-α stimulates the renin-angiotensin system through several pathways. TNF-α increases the activity of nitric oxide synthase which impairs contraction of myocytes, directly affects intracellular Ca2+ flux decreasing the inotropic response of the heart, and recruits leukocytes that can be cytotoxic to myocytes (Figure 5). All these mechanisms decrease cardiac output and lead to a cardiorenal response to increase angiotensin II activity [192,193,194]. Angiotensin II is believed to promote fibrosis by inducing the production of macrophage chemotactic protein 1 (MCP-1), a chemokine that drives migration of precursor fibroblast cells into the heart (Figure 5) [195]. Decreased TNFR1 causes decreased TGF-β activity, which is also associated with angiotensin-driven fibrosis [195]. TGF-β increases the recruitment of mast cells which release TNF-α, TGF-β, and chymase, increasing conversion of angiotensin I to angiotensin II (Figure 5) [196,197,198]. Angiotensin II receptor 1 activation connects to the TGF-β pathway by positively modulating the Wnt and β-catenin pathways for fibrosis (Figure 5) [199]. This links TNF-α to both fibroblast recruitment through angiotensin II and fibroblast maturation through TGF-β. The importance of these pathways in fibrosis is demonstrated by the improvement in heart remodeling in mice with experimental autoimmune myocarditis when given captopril or losartan, two commonly used ARBs [183,200]. TNF-α inhibitors have also been studied and used in the treatment of myocarditis and IPF, strengthening the association between TNF-α and fibrosis [201,202,203]. As mentioned previously, increased TNF-α has been observed in COVID-19 vaccine-associated myocarditis, indicating that there is risk for fibrosis even if patients appear to recover fully.

Viral myocarditis can be from either direct cardiac tissue damage or from the immune response to the virus as previously discussed. If untreated, continued cardiac dysfunction and chronic infection leads to fibrotic cardiac remodeling. The degree of remodeling can be correlated with cardiac fibrosis observed on cardiac magnetic resonance imaging (MRI) [106,161,162]. As remodeling progresses, dilated cardiomyopathy develops into heart failure as the myocardium becomes stiffer [204,205]. This process is not specific to viral myocarditis; increased fibrosis and dilated cardiomyopathy can be observed in autoimmune and ischemic cardiomyopathies [206]. In autoimmune myocarditis, chronic inflammatory cytokine and autoantibody production cause continued cardiac dysfunction and tissue damage until cardiac remodeling occurs. Even when the myocardial inflammation resolves, increased fibrosis may remain and predispose patients to later restrictive cardiomyopathies and mortality [206]. Fibrosis and scar tissue can also predispose patients to develop ventricular tachycardia and arrythmias due to aberrant conduction pathways [207]. The potential for later fatal health conditions warrants continued study and observation including COVID-19 vaccine-induced myocarditis.

## 5. Specific Considerations for Congenital Heart Defect Patients

Congenital heart defects are estimated to affect as many as 1% of live births in the US [208]. As surgical procedures have advanced, the proportion of those born with CHD who survive into adulthood has increased dramatically. As more of these patients live into adulthood with surgical correction, they are found to have decreased cardiac function compared to adults not born with these defects. Similar cardiac function as those with heart failure have been observed [6]. Previous studies found nearly 30% of hospitalized CHD patients could be attributed to heart failure symptoms. This increased risk for development of heart failure is likely from increased fibrosis [6]. CHD patients demonstrate greater risk for right heart failure due to a vast number of causes depending on the type of defect and repair. A common example is pulmonary valve regurgitation after the repair of Tetralogy of Fallot [209]. Regardless of cause, right heart failure leads to the activation of RAAS just as in traditional heart failure [210]. As previously mentioned, chronically increased angiotensin II causes cardiac fibrosis and remodeling, leading to further dilated cardiomyopathy [211]. This increased fibrosis and cardiomyopathy related to CHD becomes a comorbidity and risk factor for mortality in the setting of other serious medical conditions including severe infection.

The effects of COVID-19 infection in CHD patients are not well understood with limited data of outcomes in specific defects. Reports differ in their conclusions about the risk of COVID-19 infection severity and mortality in CHD patients [12,212,213,214,215]. Adult CHD patient mortality did increase in some studies with severity of heart disease stage, consistent with trends seen in the general population, but was not associated with the type of anatomical defect present [12,214]. One reason suggested for the lack of significant difference in outcome in adults with CHD is the availability of the COVID-19 vaccine for the adult population [216]. Other confounding factors include limited sample size, as well as heterogenicity of type and severity of CHD in adult patients [12]. As previously discussed, while not specific to myocarditis, 80% of pediatric patients from some studies had “medically complex” comorbidities, including genetic abnormalities [74]. While mortality from COVID-19 may be lower in the pediatric population, the presence of significant cardiovascular comorbidity, such as those seen in CHD, likely increases the risk of severe COVID-19 infection and death. Another study showed that pediatric CHD patients presented with more severe infection (20%) and had higher mortality (51.9%) compared with their non-CHD pediatric counterparts. While their severity of illness was lower than that of adults, this high risk for mortality was greater than both CHD and non-CHD adult COVID-19 patients [216]. This was accompanied by an increased mortality rate of 3.8% in CHD pediatric patients compared to 0.8% in non-CHD pediatric patients [216].

Similarly, the effects of the COVID-19 vaccine on adult CHD patients are not well known. One study regarding COVID-19 vaccination in 208 adult CHD patients found no increase adverse events compared to a control patient population. Of these 208 patients, only one patient developed pericarditis and recovered after standard management with non-steroidal anti-inflammatory drugs (NSAIDs) [217]. Official recommendations advocate for the use of the COVID-19 vaccine in CHD patients as the risk of severe symptoms and death from COVID-19 infection far outweighs the risk of worsened cardiac function post-vaccine. Careful observation post-vaccine is recommended for signs of cardiac symptoms in patients with CHD, and cases of myocarditis in non-CHD patients have responded well to medical management. While additional study is needed to determine the long-term effects of the COVID-19 vaccine in the CHD patient population, the fear of worsened cardiac function should not prevent vaccination in this patient population.

## 6. Conclusions

The COVID-19 pandemic is an unprecedented crisis, leading to the similarly unprecedented approval of the COVID-19 mRNA vaccines. Though the vaccines have been shown to be safe and effective, they have adverse effects, just as all vaccines do. Of particular concern has been the increasing number of reports of vaccine-associated myocarditis, of which the COVID-19 vaccines now account for the majority of reports [92]. The specific pathway involved in vaccine-associated myocarditis is dependent on the specific vaccine, but in the case of COVID-19, excessive cytokine-mediated inflammation, autoantibody formation, and dsRNA contamination have been implicated [7,166]. The inflammatory cytokines involved include TNF-α, IFN-γ, IL-6, and IL-1, with genetic predispositions to IL-6 induced inflammation believed to contribute to overactive vaccine responses [48,163]. The spike protein used in the vaccine may also cause molecular mimicry with α-myosin similar to what is observed in some COVID-19 infections [162]. Regardless of the mechanism involved, most patients have fully recovered, though rare deaths have occurred. Despite recovery, there is concern for fibrosis that persists long after myocarditis resolution [8,9]. TNF-α and TGF-β, which are elevated in COVID-19 infection and COVID-19 vaccine-associated myocarditis, are both involved in cardiac remodeling and fibrosis [10,11]. TNF-α is also involved in the activation of the RAAS system which has been implicated in worsening cardiac function and the development of cardiomyopathies [192,193]. Patients with CHD are at increased risk for cardiomyopathy even with successful repair and are prone to increased cardiac fibrosis [6]. Limited studies in CHD patients with COVID-19 have not shown any increased morbidity or mortality; similarly, there have been hardly any studies of COVID-19 vaccine-associated myocarditis in CHD patients [12]. These studies also did not account for long-term risk of fibrosis and cardiomyopathy after COVID-19 or COVID-19 vaccine-associated myocarditis in this patient population. However, what is clear from studies is that the risk of COVID-19 infection-associated myocarditis alone is significantly higher without vaccination and is more likely to lead to mortality compared to COVID-19 vaccine-associated myocarditis [68]. When taken together with the risk of severe infection and death from COVID-19 infection from non-cardiac causes, the risk of vaccine-associated adverse events is far less than infection. While the risk of myocarditis and decreased cardiac function later in life is important to consider in both CHD patients and the public, the risk of myocarditis and other serious sequelae from COVID-19 infection far outweigh the risk of myocarditis from vaccine-associated myocarditis.

## Data Availability

No new data was generated by this review.

## Glossary

ACE	angiotensin converting enzyme
ACovCS	acute COVID-19 cardiovascular syndrome
ANA	antinuclear antibody
APC	antigen presenting cell
ARB	angiotensin receptor blocker
α-SMA	alpha smooth muscle actin
ASIA	autoimmune/inflammatory syndrome induced by adjuvants
Bcl-XL	B-cell lymphoma-extra large
Be1	B-effector-1-cell
C3	complement 3
C4	complement 4
cAMP	cyclic adenosine monophosphate
CD40L	CD40 ligand
CDC	Centers for Disease Control
CHD	congenital heart defect
CTGF	connective tissue growth factor
DISC	death-inducing signaling complex
ECP	eosinophil cationic protein
HLA-DBR1	human leukocyte antigen-DBR1
HPV	human papilloma virus
ICD	implantable cardioverter-defibrillator
IFN-γ	interferon gamma
IgA	immunoglobulin A
IL-1	interleukin 1
IL-1β	interleukin 1β
IL-3	interleukin 3
IL-4	interleukin 4
IL-5	interleukin 5
IL-6	interleukin 6
IL-12	interleukin 12
IL-18	interleukin 18
IPF	idiopathic pulmonary fibrosis
ITPKC	inositol 1,4,5-triphosphate 3-kinase C
LEF	lymphoid enhancer factor
MBP	major basic protein
MCP-1	macrophage chemotactic protein 1
MIS-A	multisystem inflammatory syndrome in adults
MIS-C	multisystem inflammatory syndrome in children
MRI	magnetic resonance imaging
mRNA	messenger RNA
NFAT	calcium/nuclear factor of activated T-cells
NK	natural killer cell
NLR	NOD-like receptor
NO	nitric oxide
NOD	nucleotide oligomerization domain
NSAID	non-steroidal anti-inflammatory drug
NSP1	non-structural protein 1
NYHA	New York Heart Association
ORF3a	open reading frame 3a
ORF7	open reading frame 7
PAF	platelet-activating factor
PCR	polymerase chain reaction
PKA	protein kinase A
PRR	pattern recognition receptor
PTPN22	Protein Tyrosine Phosphatase Non-Receptor Type 22
RAAS	renin-angiotensin-aldosterone system
SARS-CoV-2	severe acute respiratory syndrome coronavirus 2
SMAD	mothers against decapentaplegic
SNP	single-nucleotide polymorphism
TAK-1	transforming growth factor-β activated kinase 1
TCF	T-cell factor
TF	tissue factor
TGF-β	transforming growth factor beta
Th1	T-helper cell 1
Th2	T-helper cell 2
Th17	T-helper cell 17
TLR	toll-like receptor
TNF-α	tumor necrosis factor alpha
TNFRI	tumor necrosis factor receptor I
TNFRII	tumor necrosis factor receptor II
Wnt	wingless-related integration site
